# Retraction: Lactosmart: a novel therapeutic molecule for antimicrobial defense

**DOI:** 10.3389/fmicb.2026.1899018

**Published:** 2026-06-09

**Authors:** Frontiers Editorial Office

The journal retracts the article cited above.

Following publication, concerns were raised regarding the scientific validity of the article. An investigation was conducted in accordance with Frontiers' policies.

The authors provided responses and supporting information; however, the journal considered that further structural characterization and validation would be required to support the conclusions. The article has been retracted.

This retraction was approved by the Chief Editors of Frontiers in Microbiology and the Chief Executive Editor of Frontiers. The authors do not agree to this retraction.

Frontiers would like to thank the concerned reader who contacted us regarding the published article.

